# Myosin II does not contribute to wound repair in *Dictyostelium* cells

**DOI:** 10.1242/bio.20149712

**Published:** 2014-09-19

**Authors:** Shigehiko Yumura, Sayaka Hashima, Satsuki Muranaka

**Affiliations:** Department of Functional Molecular Biology, Graduate School of Medicine, Yamaguchi University, Yamaguchi 753-8512, Japan

**Keywords:** Actin, Calcium ion, Myosin, Wound repair

## Abstract

Cells are always subjected to mechanical stresses, resulting in wounds of the cell membrane, but cells are able to repair and reseal their wounded membrane. Previous reports have shown that actin and myosin II accumulate around the wound and that the constriction of this purse-string closes the membrane pore. Here, we developed a microsurgical wound assay to assess wound repair in *Dictyostelium* cells. Fluorescent dye that had been incorporated into the cells leaked out for only 2–3 sec after wounding, and a GFP-derived, fluorescent Ca^2+^ sensor showed that intracellular Ca^2+^ transiently increased immediately after wounding. In the absence of external Ca^2+^, the cell failed to repair itself. During the repair process, actin accumulated at the wounded sites but myosin II did not. The wounds were repaired even in myosin II null cells to a comparable degree as the wild-type cells, suggesting that myosin II does not contribute to wound repair. Thus, the actomyosin purse-string constriction model is not a common mechanism for wound repair in eukaryotic cells, and this discrepancy may arise from the difference in cell size.

## INTRODUCTION

Cells are always subjected to mechanical or chemical damages from the environment, and often the cell membrane is wounded. In particular, cells in mechanically active tissues, such as muscle cells, are frequently wounded; however, they seem to have the ability to repair their wounded cell membrane (Abreu-Blanco et al., 2011a; Idone et al., 2008a; McNeil and Steinhardt, 2003; Sonnemann and Bement, 2011; Steinhardt, 2005). The ability to repair a membrane wound is particularly important for long-lived cells such as neurons. Inactivation of the membrane-resealing protein dysferlin can cause muscular dystrophy (Waddell et al., 2011). Plant cells also have a wound repair system (Schapire et al., 2009). Plant cells suffer from freeze-induced cell membrane wounds in winter, and their tolerance to freezing involves Ca^2+^-dependent membrane resealing. Artificial wound experiments, such as puncturing by a microneedle, ablation by intense laser illumination, or treatment with detergent, bacterial toxin, or hypotonic media have shown that mammalian cells, amphibian eggs, echinoderm eggs, fruit flies, amoebae, and budding yeast can reseal their cell membranes (Abreu-Blanco et al., 2011b; Bi et al., 1995; Gingell, 1970; Kono et al., 2012; Szubinska, 1971; Togo et al., 1999). In addition, any methods for introducing extracellular substances into the cells, including microinjection, scrape-loading, electroporation, or osmotic shock-loading, relies on the cell's ability to reseal its wounded membrane.

The immediate response to a cellular wound is to plug the cell membrane pores, thereby avoiding an influx of extracellular molecules and preventing the loss of cytoplasm. The membrane pores are rapidly closed by the recruitment and fusion of internal vesicular membrane to the cell membrane. During repair, both the cell membrane and the underlying cortical cytoskeleton restore their functions at the wound site. A common feature of wounding is the entry of Ca^2+^ from the wounded site, which triggers a quick repair response (Idone et al., 2008b; McNeil et al., 2003; McNeil, 2002; Reddy et al., 2001).

When *Xenopus* oocytes are punctured with a glass needle, the circular wound circumferentially constricts coincident with the recruitment of actin and myosin II to the edge of the wound (Bement et al., 1999). It has been suggested that the constriction of the actomyosin purse-string helps to close the wound (Darenfed and Mandato, 2005). Contractile actomyosin purse-strings generally appear in the cleavage furrow of dividing cells (Schroeder, 1973; Yumura and Uyeda, 2003; Yumura et al., 1984), in the apical region of epithelial cells during embryonic morphogenesis (Sawyer et al., 2010), and at the edge of tissue wounds (Abreu-Blanco et al., 2011b). In fibroblasts, knockdown of myosin IIB suppresses wound-induced exocytosis and the membrane resealing process. Knockdown of myosin IIA has no inhibitory effect on resealing of initial wounds but inhibits the facilitated rate of resealing that is normally found at wounds repeatedly made at the same site (Togo and Steinhardt, 2004).

In the present study, we investigated wound repair in the cellular slime mold, *Dictyostelium discoideum*. *Dictyostelium*, as a model organism, has been used to study cell migration, cell division, and related cytoskeletons. Various mutant cells of actin-binding proteins, including myosin II null cells, are available, which is an advantage for the present study. If actin and myosin II accumulate and form actomyosin purse-strings at wound sites in *Dictyostelium* cells, *Dictyostelium* will be a powerful model for research on contractile rings. When *Dictyostelium* cells are cut in half, the nucleated fragments resume normal migration within seconds (Swanson and Taylor, 1982), suggesting that *Dictyostelium* cells are also capable of wound repair. In the present study, when *Dictyostelium* cells were wounded by partial cutting with a microneedle, they immediately repaired the wound. During this process, actin accumulated at the wound site, but myosin II did not. The wounds were repaired in myosin II null cells to a comparable degree as in wild-type cells, suggesting that myosin II does not contribute to wound repair. Therefore, the constriction of the actomyosin purse-string is not applicable to wound repair in *Dictyostelium* cells, and it is not a common mechanism for wound repair in eukaryotic cells. The reason for this discrepancy will be discussed.

## RESULTS

### Wound repair after microsurgery

**Fig. 1.**
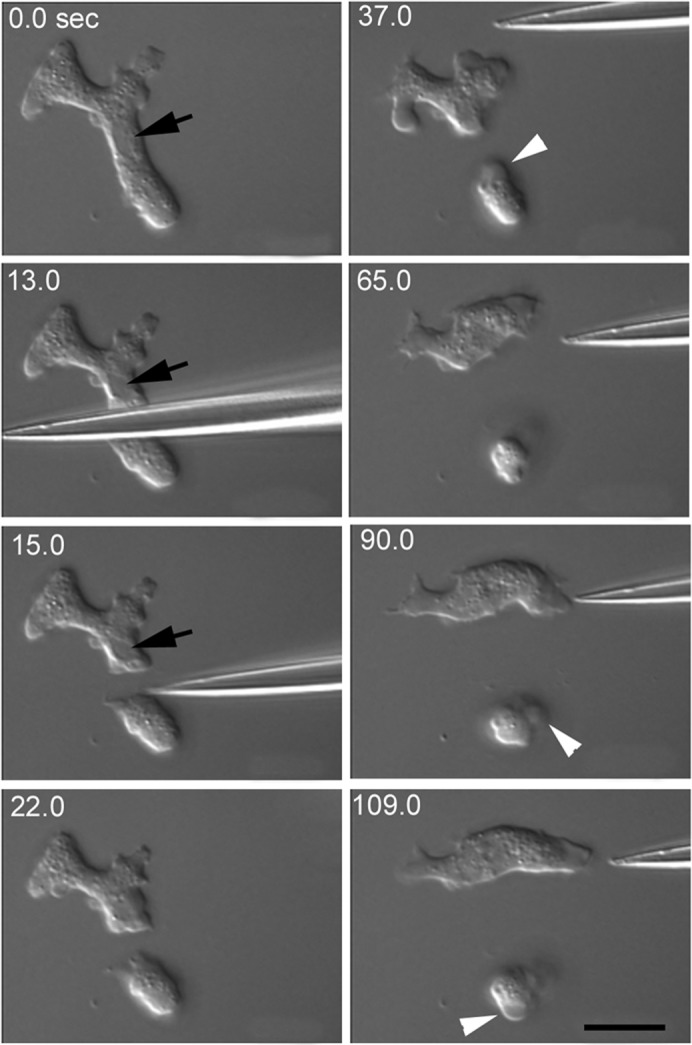
Cells can repair wounds. Differential interference contrast microscopy showing that a *Dictyostelium* cell was cut by a microcapillary containing 10 µM cAMP. After cutting, the fragment containing a nucleus (arrow) could migrate normally and even showed chemotaxis when cAMP was locally applied (37–109 sec). The enucleated fragment did not show chemotaxis toward cAMP and repeatedly extended blebs (arrowheads). Scale bar: 10 µm.

Swanson and Taylor showed that *Dictyostelium* cells can be cut in half and that the nucleated fragments resume normal migration within seconds, suggesting that *Dictyostelium* cells have a powerful wound repair mechanism (Swanson and Taylor, 1982). First, we attempted to confirm this observation. Fig. 1 shows a typical microsurgery experiment, where a migrating cell was cut by a microcapillary tube containing cAMP, a chemoattractant of this organism. The fragment containing a nucleus (arrow) could migrate normally and even showed chemotaxis when cAMP was locally applied (37–109 sec). Thus, *Dictyostelium* cells can repair their wounded cell membrane.

**Fig. 2.**
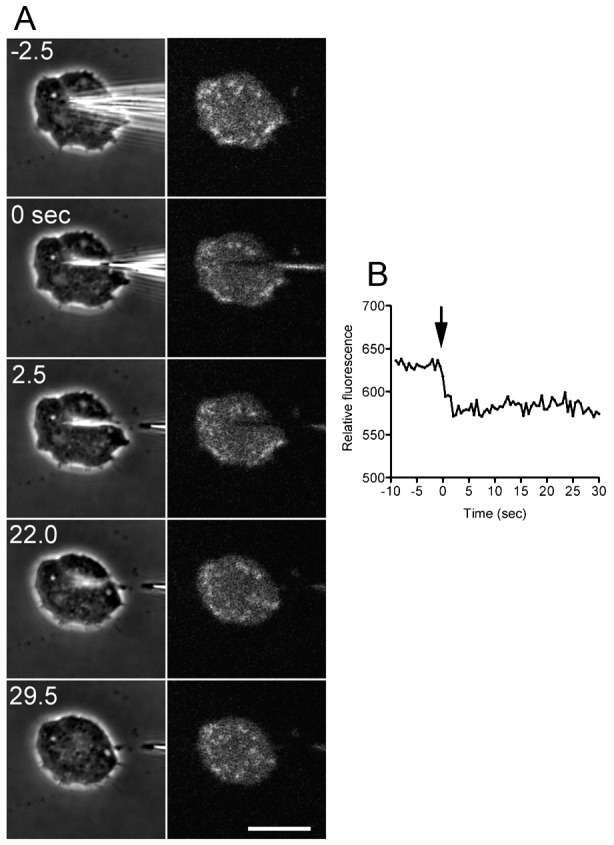
Leaks of fluorescent dye after wounding. The cells were loaded with CytoRed in advance. (A) A representative time course of phase-contrast and fluorescence images. After cutting, the fluorescence intensity immediately decreased, indicating that CytoRed leaked out of the cell. (B) A time course of total fluorescence intensity of the cytoplasm. The leak was ceased within only 3.5±1.2 sec (n = 12) sec after cutting, indicating that the membrane pore was closed by this time. The arrow shows the time the cell was cut. Scale bar: 10 µm.

Next, a microneedle was applied to make a small wound onto the cell body, which was visualized using confocal microscopy. The cells had been previously incubated with CytoRed, which, once esterified, became membrane impermeable after being incorporated into the cells and showed a diffuse distribution in the cytoplasm. After cutting, the fluorescence intensity immediately decreased (Fig. 2A), indicating that the cytoplasm containing CytoRed leaked out of the cell. The leak was ceased within only 3.5±1.2 sec (n = 12) after cutting (Fig. 2B), indicating that the membrane pore was closed by this time. In earlier experiments, we attempted to wound the cell by poking it with a microneedle, as is performed for other cells such as animal eggs and fibroblasts, but this was difficult because the cell size was much smaller (less than 10 µm in diameter) and because the cells actively migrated without rigid adhesion to the substratum.

### Ca^2+^ is essential for wound repair

**Fig. 3.**
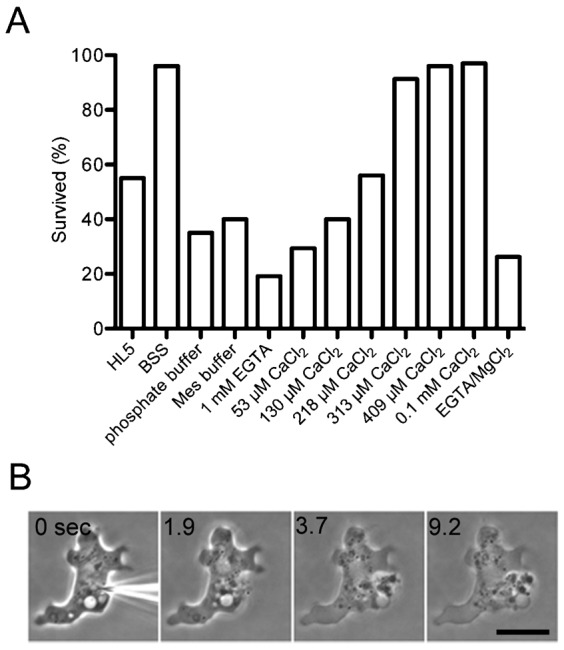
Effects of divalent cations in the extracellular solution. (A) The survival rate of the cells after cutting was examined under the indicated salt or buffer conditions. Calcium was not included in the composition of HL5 medium but might contain submillimolar concentrations of Ca^2+^. BSS contains 3 mM CaCl_2_. The free Ca^2+^ level (53, 130, 218, 313, 409 µM) was adjusted by the Ca–EGTA buffer, as described in Materials and Methods. All solutions, except HL5 and the phosphate buffer, contained 5 mM MES buffer. The survival rates of more than 20 cells under each condition were examined. Note that concentrations higher than 313 µM Ca^2+^ are required for >90% survival and that Mg^2+^ cannot be substituted for Ca^2+^. (B) A representative phase-contrast images of cell rupture after wounding in the absence of Ca^2+^. Note that the cytoplasm was expelled out of the cell after cutting. Scale bar: 10 µm.

Previous reports on the wound repair of animal eggs and cultured cells have shown that external Ca^2+^ is essential for wound repair (Bi et al., 1995; Steinhardt et al., 1994; Steinhardt, 2005). Thus, wound repair in *Dictyostelium* cells when changing the external Ca^2+^ concentration was evaluated. In BSS containing 3 mM CaCl_2_, a physiological buffered salt solution for these cells, nearly all of the wounds (96%, n = 50) were repaired after cutting (Fig. 3A). In the absence of Ca^2+^ (1 mM EGTA), 82% of the cells (n = 50) failed to repair themselves and finally ruptured (Fig. 3B). The cells were then wounded in the presence of varied free Ca^2+^ concentrations, which were adjusted using Ca–EGTA buffer. Fig. 3A shows that higher than 0.3 mM Ca^2+^ is required for efficient repair. When CaCl_2_ was replaced with MgCl_2_, another divalent cation, the wound could not be repaired, suggesting that external Ca^2+^ is essential for wound repair.

### Intracellular Ca^2+^ increased immediately after cutting

**Fig. 4.**
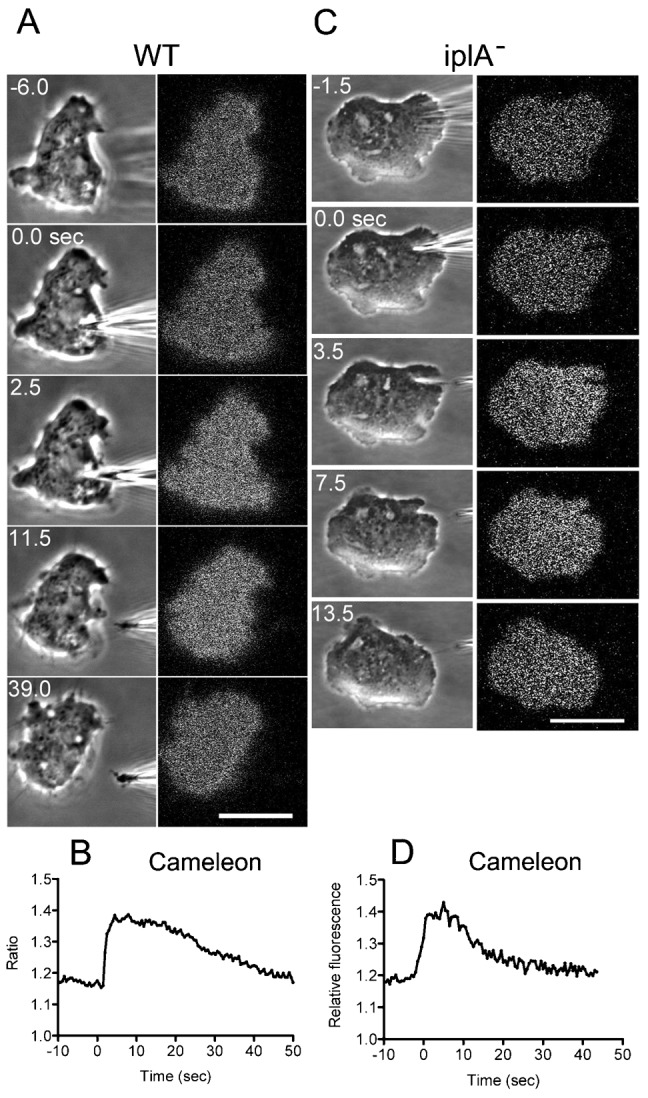
Transient increase of intracellular Ca^2+^ after wounding. (A) Representative phase-contrast and fluorescence ratio images of a wild-type cell expressing Cameleon-YC-Nano15 during the wound assay. (B) A time course of the fluorescence ratio (Venus/CFP) of the cytoplasm is plotted. Note that the intracellular Ca^2+^ concentration increased transiently. (C) Representative phase and fluorescence ratio images of an *iplA* null cell expressing Cameleon-YC-Nano15 during the wound assay. (D) A time course of the fluorescence ratio (Venus/CFP) of the cytoplasm of the *iplA* null cell is plotted. Note that the intracellular Ca^2+^ concentration transiently increased in a similar manner to wild-type cells. Scale bars: 10 µm.

To examine whether Ca^2+^ enters the cytoplasm after wounding, Cameleon-YC-Nano15, a GFP(green fluorescent protein)-based FRET sensor for Ca^2+^, was expressed in the cells. Cameleon-YC-Nano15 is a synthetic construct composed of ECFP, calmodulin, the calmodulin-binding domain of myosin light chain kinase (M13), and Venus (Horikawa et al., 2010). Ratio images of the fluorescence (Venus/ECFP) are shown in Fig. 4A,B and indicate that the intracellular Ca^2+^ immediately increases with a peak at 4.9±2.8 sec and decreases at 44.5±10.4 sec after cutting (n = 32). We did not observe that Ca^2+^ levels increased locally at the wound site and then propagated to the cytoplasm, most likely because the diffusion rate of Ca^2+^ is too fast for detection.

The increase in Ca^2+^ is most likely caused by influx of Ca^2+^ from the outside of the cell but may be caused by the release of Ca^2+^ from an intracellular stock such as the endoplasmic reticulum. Therefore, Cameleon-YC-Nano15 was expressed in cells lacking the *iplA* gene, which is an inositol 1,4,5-trisphosphate (InsP(3)) receptor-like gene. These mutant cells show no increase in Ca^2+^ after chemotactic stimulation (Traynor et al., 2000). After cutting, the Ca^2+^ level increased to a degree comparable to that in wild-type cells (Fig. 4C,D), indicating that the observed transient increase in Ca^2+^ is not caused by its release from the intracellular stock but by the influx from the outside through the wound pore.

### Actin but not myosin II accumulates at the wound site

**Fig. 5.**
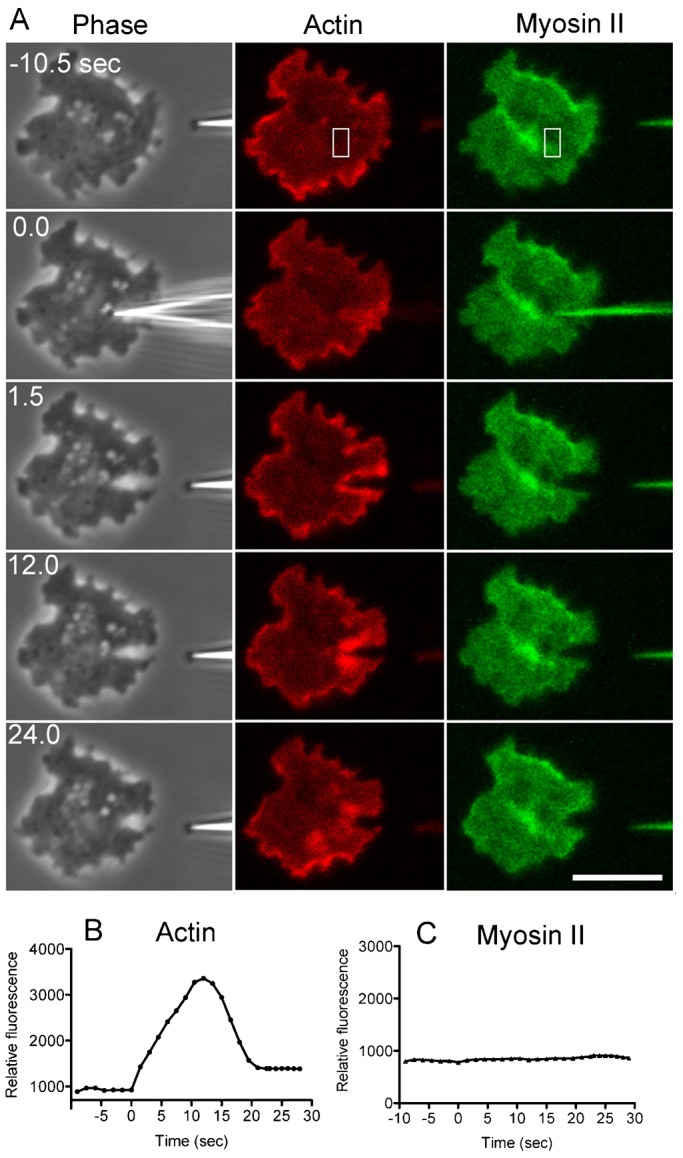
Dynamics of actin and myosin II after wounding. Cells simultaneously expressing mCherry-lifeact and GFP myosin II were cut by a microneedle and visualized by confocal microscopy. (A) The phase-contrast images (left), the fluorescence of mCherry-lifeact, a marker of actin filaments (middle), and the fluorescence of GFP-myosin II (right) are shown over time during wound repair. Actin filaments clearly accumulated at the wound site but myosin II did not. (B) A time course of the fluorescence intensity of mCherry-lifeact at the wound site. (C) A time course of the fluorescence intensity of GFP-myosin II at the wound site. The fluorescence intensities in the rectangles in panel A were measured over time. Note that myosin II did not accumulate at the wound site. Scale bar: 10 µm.

**Fig. 6.**
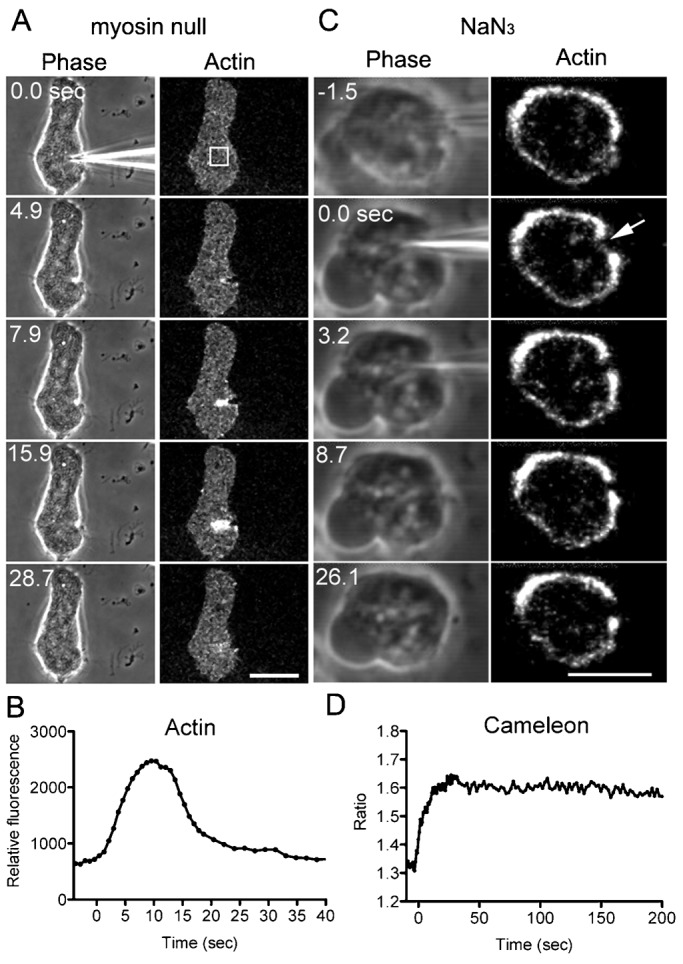
Actin accumulation is myosin II-independent and energy-dependent. (A) Myosin II null cells expressing GFP-lifeact were cut by a microneedle and visualized by confocal microscopy. A typical time course of phase-contrast (left) and fluorescence (right) images of the cells during wound repair. Note that actin filaments accumulated in a manner comparable to those in wild-type cells. (B) A time course of the fluorescence intensity of GFP-lifeact at the wound site. The fluorescence intensity in the rectangle in panel A was measured over time. (C) In the presence of 1 mM sodium azide, wild-type cells expressing GFP-lifeact were cut by a microneedle. A typical time course of phase-contrast (left) and fluorescence (right) images of the cells in the presence of sodium azide during wound repair. Note that actin filaments did not accumulate at the wound site (arrow). (D) The fluorescence ratio (Venus/CFP) of the cytoplasm of wild-type cells expressing Cameleon-YC-Nano15 in the presence of sodium azide was plotted over time. Note that the intracellular Ca^2+^ concentration did not return to the basal level after its increase was induced by cutting. Scale bars: 10 µm.

Actin and myosin II have been reported to accumulate at the wound site in several types of animal eggs and cultured cells (Abreu-Blanco et al., 2012b; Mandato and Bement, 2001; Miyake et al., 2001). When *Dictyostelium* cells simultaneously expressing mCherry-lifeact, a reporter of actin filaments, and GFP-myosin II were cut by a microneedle, actin vigorously accumulated at the wound sites. Actin began to accumulate at 2.8±1.3 sec, reached a peak at 8.2±2.1 sec, and lasted 22.6±8.1 sec (n = 25) until returning to its resting level (Fig. 5A,B). When cells were cut into two fragments, as shown in Fig. 1, actin also accumulated at the wound edges (data not shown). On the other hand, no myosin II accumulated at the wound sites (Fig. 5A,C). When myosin II null cells were cut, nearly all of the mutant cells repaired the wounds (92.3%, n = 52), and this reaction was comparable to the wound repair of wild-type cells (96%, n = 51, Fig. 3A). In addition, actin filaments accumulated at the wound site in myosin II null cells in a manner comparable to that in wild-type cells (Fig. 6A,B). Therefore, myosin II is not required for wound repair, suggesting that the purse-string model of actomyosin is not applicable to wound repair in *Dictyostelium* cells. Incidentally, when a single cell was repeatedly cut, actin accumulated at the wound site in the same way at each time (data not shown).

### Actin accumulation at wound sites is energy-dependent

Cells were cut by a microneedle in the presence of sodium azide, an inhibitor of ATP synthesis. Immediately after incubation with 1 mM sodium azide, cells stopped migration and became round. Most of the wounded wild-type cells did not rupture in the presence of sodium azide after cutting. Interestingly, actin filaments did not accumulate at the wound sites (Fig. 6C). The concentration of intracellular Ca^2+^ increased after cutting and did not decrease for a long time afterward (Fig. 6D). When sodium azide was removed from the external solution, the wounded cells resumed migration. Thus, actin accumulation is energy-dependent, and membrane repair is incomplete without energy. However, that the influx of Ca^2+^ stopped increasing in the same way as that of untreated cells suggests that the membrane pore was almost closed by this time. These experiments indicate that the accumulation of actin at wound sites is not essential.

### Membrane repair in the absence of actin filaments

**Fig. 7.**
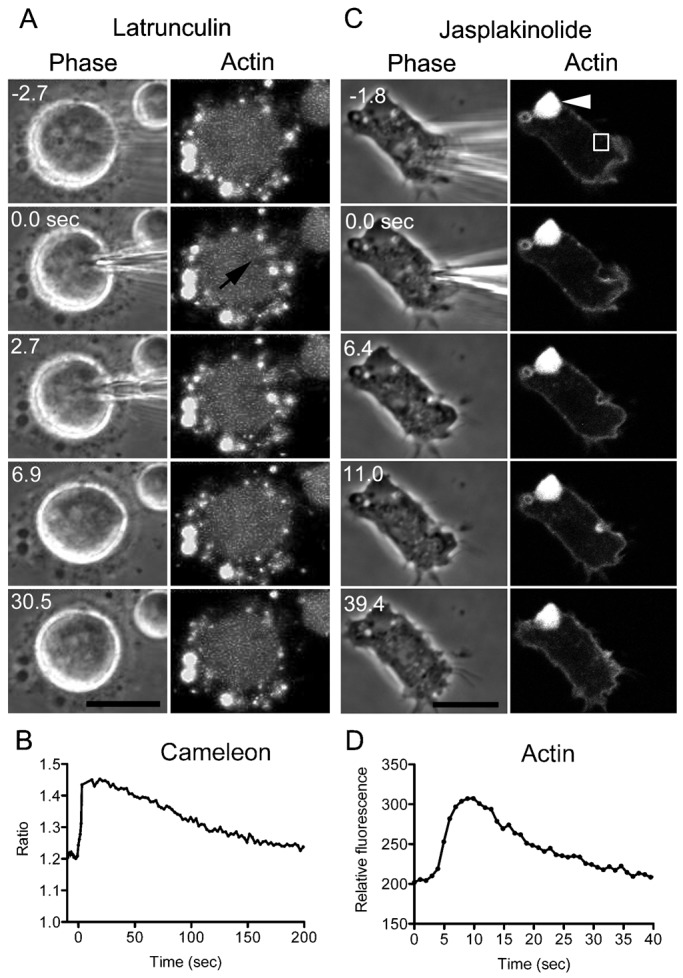
Wound repair in the absence of actin filaments. Wild-type cells expressing GFP-lifeact were cut in the presence of latrunculin B. Several small spheres containing actin remained around the cells while the cells became round, but there were no specific actin structures inside the cells. (A) A typical time course of phase-contrast (left) and fluorescent (right) images during wound repair. Note that GFP-lifeact did not accumulate at the wound site. (B) The fluorescence ratio (Venus/ECFP) of the cytoplasm of a wild-type cell expressing Cameleon-YC-Nano15 in the presence of latrunculin B was plotted over time. Note that the intracellular Ca^2+^ concentration immediately increased after cutting and then slowly returned to the basal level. (C) Wild-type cells expressing GFP-lifeact were cut in the presence of jasplakinolide. A typical time course of phase-contrast (left) and fluorescent (right) images during wound repair. The arrowhead shows the aggregate of actin filaments, which is typical in cells treated with jasplakinolide. (D) A time course of the fluorescence intensity of GFP-lifeact at the wound site. The fluorescence intensity in the rectangle in panel C was measured over time. Note that actin filaments accumulated at the wound sites, but the peak time and the lasting time were significantly longer than those of untreated cells. Scale bars: 10 µm.

To further examine whether the accumulation of actin filaments is required for wound repair, cells expressing GFP-lifeact were cut in the presence of latrunculin B, a depolymerizer of actin filaments. Upon incubation with latrunculin B, the cells became round, and actin-containing structures such as pseudopods disappeared (Fig. 7A). Several small spheres containing actin remained outside the cells while the cells became round, but there were no actin-specific structures within the cells. The cells became softer in the presence of latrunculin B but did not rupture when cut. GFP-lifeact did not accumulate at the wound sites in the treated cells (Fig. 7A). When latrunculin B was removed by exchanging the external solution, the cells eventually resumed migration.

When cells expressing Cameleon-YC-Nano15 were cut in the presence of latrunculin B, the intracellular Ca^2+^ level immediately increased, but it took a longer time to return to the resting level (>200 sec, Fig. 7B). Therefore, the cells took a longer time to repair the wound in the absence of actin accumulation.

Next, the cells were cut in the presence of jasplakinolide, a membrane-permeable, actin filament stabilizer. When the cells were incubated in the presence of jasplakinolide, a large aggregate (arrowhead in Fig. 7C) or several aggregates of actin filaments were found in each cell, as previously reported (Lee et al., 1998). When the cells were cut in the presence of jasplakinolide, actin accumulated at the wound sites, but the peak time (10.8±1.6 sec, n = 25) and duration (34.4±5.0 sec, n = 25) were significantly longer than those of untreated cells, most likely due to stabilization of actin filaments or a decrease in the available monomeric actin due to jasplakinolide (Fig. 7C,D). However, most of the cut cells did not rupture (98/100).

These observations indicate that the accumulation of actin is not essential for wound repair but is required for proper wound repair.

## DISCUSSION

We developed a microsurgical wound assay in *Dictyostelium* cells. Here, actin accumulated at the wound sites but myosin II did not. In addition, myosin II null cells repaired the wound in a way comparable to wild-type cells. Therefore, myosin II is not required for wound repair in *Dictyostelium* cells. Previously, in *Xenopus* eggs and *Drosophila* embryos, a purse-string model of actomyosin was proposed for closing wounded membrane pores (Abreu-Blanco et al., 2012b; Mandato and Bement, 2001). However, based on the present study, this model is not applicable to wound repair in *Dictyostelium* cells. Similarly, no accumulation of myosin II to wound sites has been reported in fibroblasts and yeast, although the purse-string model is not mentioned (Kono et al., 2012; Togo and Steinhardt, 2004). This discrepancy may be caused by the differences in the sizes of the cells. Lager cells suffer from a larger force of gravity, which affects the tension of the cell membrane. In fact, when *Xenopus* eggs are denuded of their fertilization envelopes, they deform from a spherical shape due to gravity (Yoneda et al., 1982). The wounded cell membrane sites are then forced to open due to the force of gravity. Therefore, a larger force generated by the myosin II motor is necessary to counteract this force. As another explanation, the sizes of the wounds were different in the organisms submitted to the wound assay: 100–200 µm for *Xenopus* eggs, and 20–50 µm for *Drosophila* embryos. The sizes of the wounds in this study were as large as 1–2 µm; therefore, larger wounds might require more power. However, even when a single *Dictyostelium* cell was cut into two fragments (the wound size was the maximum for these cells), no myosin II accumulated to the wound sites (data not shown).

Interestingly, in the *Xenopus* experiments, myosin II accumulated at the wound sites in the presence of latrunculin B (Bement et al., 1999), which indicates that myosin II can accumulate independent of actin filaments. Therefore, the accumulation of myosin II and actin may be considered separately.

Outstanding imaging techniques have shown that cortical actin filaments flowed toward the wound edge in *Xenopus* eggs (Mandato and Bement, 2001). We also attempted to observe a flow of actin filaments to the wound sites using total internal reflection fluorescence microscopy (data not shown). Although we were able to observe individual filaments in the cortex (Yumura et al., 2008; Yumura et al., 2013), we did not detect such flow to the wound sites. Most likely, actin seems to accumulate by de novo assembly in *Dictyostelium* cells. Our preliminary observation showed that the Arp2/3 complex and cofilin accumulated at the wound site, supporting this idea. Jasplakinolide retarded the accumulation of actin at the wound site, and this can be explained by limited available monomeric actin or by changes in the stabilization of actin filament dynamics in the presence of jasplakinolide.

The accumulation of actin filaments at wound sites has been reported in various cells, but the processes are variable among different cells. In epithelial cells, actin shows a two-phase response. Initially, actin rapidly disassembles at the wound sites after wounding and then accumulates with a peak at approximately 100 sec (Godin et al., 2011). This disassembly is considered to facilitate the access and fusion of small intracellular membrane vesicles to the wounded cell membrane. However, we could not detect any disassembly prior to actin accumulation in *Dictyostelium* cells. Actin is first detected at the wound sites within approximately 3 sec after wounding in *Dictyostelium* cells, but this occurs at 30 sec in *Xenopus* eggs (Bement et al., 1999), at 30 sec in *Drosophila* (Abreu-Blanco et al., 2011b), and at 22 sec in epithelial cells (Godin et al., 2011). This very fast response of actin accumulation in *Dictyostelium* may hinder our observation of disassembly immediately after wounding.

Intracellular signals for wound repair have been investigated. The influx of Ca^2+^ after wounding has been reported in various cells, including nerve cells (Rehder et al., 1992), sea urchin eggs (Steinhardt et al., 1994; Terasaki et al., 1997), and fibroblasts (Steinhardt et al., 1994; Togo et al., 1999). This influx of Ca^2+^ may be a trigger for wound repair, including membrane plug formation and rearrangement of the cytoskeleton. In the present study, the measurement of intracellular Ca^2+^ using a FRET-based calcium sensor revealed that Ca^2+^ increased immediately after wounding of *Dictyostelium* cells. In addition, Ca^2+^ increased in *iplA* null cells after wounding in a manner comparable to that of wild-type cells. Thus, the observed, transient increase in Ca^2+^ is not caused by release from the intracellular stock but by influx from the outside through the wound pores. When a calcium ionophore is applied to *Dictyostelium* cells, actin transiently assembles at the cortex, suggesting that the entry of Ca^2+^ can be a trigger for actin assembly (Yumura, 1993; Yumura, 1994). Cells failed to repair the wound in the presence of a lower concentration of external Ca^2+^, and concentrations higher than 0.3 mM Ca^2+^ outside the cells was required for wound repair. Most likely, the influx of Ca^2+^ is required for membrane resealing, which is common in wound repair processes in other organisms (Bement et al., 1999; Steinhardt et al., 1994; Togo et al., 1999).

In addition to Ca^2+^, a number of intracellular signals are known to regulate actomyosin assembly. Rho and Cdc42, which are small G proteins, are activated at wound sites in a Ca^2+^-dependent manner, which directs actomyosin assembly in *Xenopus* eggs (Benink and Bement, 2005). Protein kinase C is also involved in remodeling of the cytoskeleton in *Xenopus* eggs (Bement and Capco, 1990) and budding yeasts (Kono et al., 2012). Many mutants deficient in intracellular signals in *Dictyostelium* cells are available; therefore, we will be able to clarify the signal cascade in wound repair using this powerful system. The recent exhaustive proteome analysis of wound repair also gives new insight into this field (Mellgren, 2010).

Energy depletion by treating cells with sodium azide decreased the efficiency of their repair. The intracellular Ca^2+^ concentration remained at a higher level after the initial increase after wounding in the presence of sodium azide, but it decreased shortly after the peak in untreated cells. Therefore, as a simple interpretation, the pumping out of Ca^2+^ from the cytoplasm may be hindered by the energy depletion. The accumulation of actin at the wound sites was also hindered by the energy depletion. The prolonged, non-physiological higher level of Ca^2+^ or low level of energy may disturb new assembly of actin filaments. Otherwise, actin accumulation may directly facilitate pumping out of Ca^2+^, as discussed later.

**Fig. 8.**
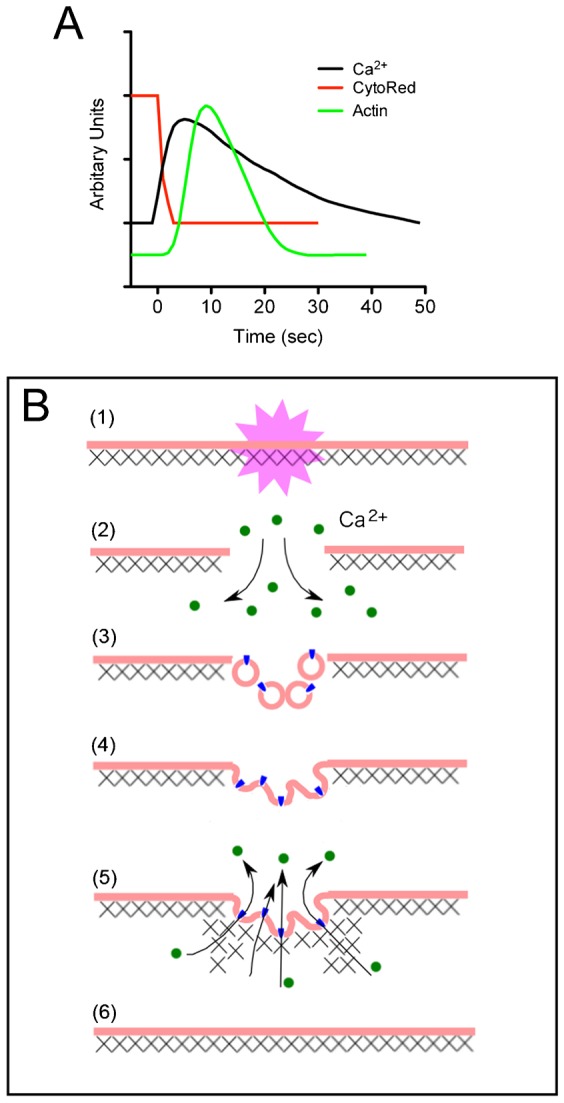
Current model for wound repair. (A) Comparison of the time courses for the influx of Ca^2+^, efflux of CytoRed and dynamics of actin during wound repair. Each graph was normalized and plotted based on averaged results of multiple experiments. (B) The current model. There are two steps in wound repair: actin-independent (2–4) and actin-dependent (5). In the first step, when the cell is wounded (1), Ca^2+^ (green particles) enters the cytoplasm from outside the cell via the wound membrane pore (2). The influx of Ca^2+^ triggers the accumulation of small intracellular vesicles and fusion to the wound membrane (3–4). The second step is dependent on actin assembly to secure the repair (5). This actin assembly is triggered by the influx of Ca^2+^ and is independent of myosin II. Myosin II does not contribute to membrane resealing. Actin filaments serve as the mechanical supports to prevent the wound from expanding further. In addition, the accumulated actin enhances the activity of Ca^2+^ pumps (blue particles) to pump Ca^2+^ out of the cell, resulting in return to the resting Ca^2+^ level in the cytosol. The accumulated actin filaments disassemble, and finally wound repair is completed (6). Crosses depict the actin filaments.

Even in the presence of latrunculin B, the cells could repair the wounds to some extent. Therefore, the accumulation of actin is not essential for wound repair. Red blood cells and liposomes can reseal their membrane pores after rupture due to osmotic shock or electroporation (Chang and Reese, 1990; Kanda et al., 1979; Karatekin et al., 2003). Therefore, cell membranes have an intrinsic ability to autonomously reseal its broken sites. Most likely, the accumulation of actin filaments has a subtle role in membrane resealing. The following roles of actin have been discussed previously: 1) actin serves as the mechanical support to prevent the wound from expanding further, and 2) actin carries materials such as small vesicles to repair or fill the wound pores (Abreu-Blanco et al., 2012a). From the present results, the release of CytoRed and entry of Ca^2+^ ended within 3–5 sec after cutting. Thus, the wounded pores were almost closed before this time. The accumulation of actin began approximately 3 sec after the cutting and peaked at approximately 10 sec (Fig. 8A). Thus, the second role of actin is unlikely.

Membrane resealing involves fusion of the intracellular vesicles to the wounded membrane. When fibroblasts whose cell membranes were stained with fluorescent lipophilic dye were wounded, the fluorescence rapidly decreased at the wounded sites, suggesting that new membrane is inserted from the intracellular region (Togo et al., 1999). Most likely, Ca^2+^ triggers the fusion of intracellular vesicles to the cell membrane, which is reminiscent of the Ca^2+^-dependent exocytosis of synaptic vesicles to release neurotransmitters. In this case, the kinesin motor family contributes to the trafficking of synaptic vesicles (Bloom, 2001). Microtubules are also involved in wound repair (Mandato and Bement, 2003), but their role in this process remains to be clarified in future studies.

The observation that the recovery of Ca^2+^ in the presence of latrunculin B required more time suggests that the accumulation of actin at the wound site is responsible for pumping Ca^2+^ out of the cell. Recent studies have shown that the activities of the Ca^2+^ pump in the cell membrane are activated by actin filaments but not by monomeric actin in human erythrocytes (Dalghi et al., 2013).

Our present model for wound repair in *Dictyostelium* cells is depicted in Fig. 8B. There are two steps in wound repair: actin-independent and actin-dependent. In the first step, when the cell is wounded, Ca^2+^ enters the cytoplasm from the outside via the wound membrane pore. The influx of Ca^2+^ triggers the accumulation of small intracellular vesicles that fuse with the wound membrane. The second step is dependent on the accumulation of actin to secure the repair, and actin may serve as the mechanical support to prevent the wound from expanding further. In addition, the accumulated actin may enhance the activity of the Ca^2+^ pump for rapid recovery to the resting level in the cytosol. Myosin II does not contribute to wound repair.

In future work, the type of signals and other components that are required for membrane resealing as well as the mechanism that contributes to the accumulation of actin and membrane resealing must be determined. The present wound assay also provides an excellent model system for investigating how actin assembly is locally regulated, which is still an unsolved question in understanding the molecular mechanism of cell migration and cytokinesis.

## MATERIALS AND METHODS

### Cell strains and culture

*Dictyostelium discoideum* cells were cultured at 22°C in a plastic dish containing HL5 medium (1.3% bacteriological peptone, 0.75% yeast extract, 85.5 mM D-glucose, 3.5 mM Na_2_HPO_4_^.^12 H_2_O, 3.5 mM KH_2_PO_4_, pH 6.3). Plasmid DNA constructs, including Cameleon-YC-Nano15, mCherry-lifeact, GFP-lifeact, and mRFP-myosin II, were transformed in cells by electroporation, as described previously (Yumura et al., 1995). Cameleon-YC-Nano15 was constructed by Horikawa et al. (Horikawa et al., 2010). mCherry-lifeact, GFP-lifeact and mRFP-myosin II were constructed by N. Umeki and T. Q. P. Uyeda. Cells transformed with the plasmid DNA were selected in 10 ml HL5 medium containing 10 µg/ml G418 (Sigma) and/or 10 µg/ml blasticidin (Kaken) in plastic dishes. The cells were washed with BSS (10 mM NaCl, 10 mM KCl, 3 mM CaCl_2_, and 2 mM MES, pH 6.3) and incubated in the same solution for 3–5 hrs before the experiments.

To incorporate the fluorescent dye into the cells, the cells were incubated with a final concentration of 10 µM CyoRed (7-Isobutyloxycarbonyloxy-3H-phenoxazin-3-one, Dojindo) for 5 min and then washed three times with BSS by centrifugation. The dye was hydrolyzed by an endogenous esterase after incorporation. To observe the fluorescence of CytoRed, the excitation wavelength was set at 543 nm, and the emission wavelength was selected by a barrier filter (>590 nm) in a confocal microscope (LSM 510 Meta, Zeiss).

### Microsurgery and microscopy

Microneedles or tapered microcapillaries were made by a puller (PG-1, Narishige, Japan) and a micro-forge (MF-900, Narishige) from glass rods (G-1000, Narishige) or microcapillaries (GD-1, Narishige), respectively. The needles were then mounted to a manipulator (3Man, S Company) attached to an inverted microscope (IX71, Olympus) or a confocal microscope (LSM 510 Meta, Zeiss). The cells were placed in a glass-bottom dish, and the fluorescences due to GFP and mCherry were excited by the 488-nm line of an argon laser and the 543-nm line of a HeNe laser, respectively. Time-lapse images were acquired at an interval of 0.2–1 sec. The objective lens (×100, Plan Neofluar, NA 1.3) was mainly used.

To examine the intracellular Ca^2+^ concentration, cells expressing Cameleon-YC-Nano15 were observed using confocal microscopy. Fluorescence images of the ECFP and Venus signals were acquired by setting the excitation (458-nm line of an argon laser) and emission (the 475–525-nm filter set for ECFP and the 515–530-nm filter set for Venus). The free Ca^2+^ concentration was calculated using the Ca–EGTA Calculator (http://maxchelator.stanford.edu/CaEGTA-TS.htm). The Ca–EGTA buffer contained 3 mM Mes (pH 6.3), 1 mM EGTA and various concentrations of CaCl_2_, to make a series of free Ca^2+^ buffers ranging from 53 µM to 409 µM.

Latrunculin B (Sigma) was dissolved in dimethyl sulfoxide (DMSO) to make a stock solution of 2 mM, and the final solution used in the experiments consisted of 5 µM latrunculin B in BSS. Jasplakinolide (Sigma) was dissolved in DMSO to make a stock solution of 0.5 mM, and the final solution used in the experiments consisted of 8 µM jasplakinolide in BSS.

Thirty minutes after incubation in the presence of latrunculin B or jasplakinolide, the cells were wounded and visualized by confocal microscopy. As control experiments, the addition of only DMSO (0.25%) did not affect the cell morphology or wound repair. Sodium azide was dissolved in distilled water at 100 mM, and a final solution of 1 mM sodium azide in BSS was used for the experiments.

### Image analysis

The time course of fluorescence intensity was analyzed, and the ratios of the images (Venus/ECFP) were calculated using Image J (http://rsbweb.nih.gov/ij).
